# Highly Stretchable Thermoplastic Polyurethane Separators for Li-Ion Batteries Based on Non-Solvent-Induced Phase Separation Method

**DOI:** 10.3390/polym16030357

**Published:** 2024-01-29

**Authors:** Tae Hyung Kim, MinSu Kim, Eun Ji Kim, Minu Ju, Ji Soo Kim, Seung Hee Lee

**Affiliations:** 1 Department of Nano Convergence Engineering, Jeonbuk National University, Jeonju 54896, Republic of Korea; kth1127@jbnu.ac.kr (T.H.K.); minsu.kim@jbnu.ac.kr (M.K.); jie1230@naver.com (E.J.K.); wer9875@gmail.com (M.J.); 2 Department of JBNU-KIST Industry-Academia Convergence Research, Jeonbuk National University, Jeonju 54896, Republic of Korea; rlawltn5006@naver.com; 3 Department of Polymer-Nano Science and Technology, Jeonbuk National University, Jeonju 54896, Republic of Korea

**Keywords:** polymeric membranes, stretchable membranes, porous membranes, non-solvent induced phase separation

## Abstract

The growing interest in wearable and portable devices has stimulated the need for flexible and stretchable lithium-ion batteries (LiBs). A crucial component in these batteries is the separator, which provides a pathway for Li-ion transfer and prevents electrode contact. In a flexible and stretchable LiB, the separator must exhibit stretchability and elasticity akin to its existing counterparts. Here, we developed a non-modified thermoplastic polyurethane (TPU) separator using the non-solvent induced phase separation (NIPS) technique. We compared their performance with commercially available polypropylene (PP) separators. Our results demonstrate that TPU separators exhibit superior elasticity based on repeated stretch/release tests with excellent thermal stability and electrolyte wettability. Furthermore, our findings confirm that TPU separators, even after being repeatedly stretched and released, can function effectively without severe damage in a fabricated coin cell LiB with high oxidative stability, as evidenced by linear sweep voltammetry, like commercially available separators.

## 1. Introduction

The recent emergence of wearable and portable devices, characterized by flexibility, rollability, and lightness, has brought about significant innovations in fields such as healthcare devices [1,2], artificial skin [3,4], and telecommunications [5], offering endless possibilities for exploring new markets. These devices demand a substantial amount of energy and a stable power supply due to prolonged outdoor activities. To ensure the stable operation of such devices, there is growing interest in implementing stretchable energy storages with forms or structures that can adapt to contracting and expanding areas, such as clothing or skin [6,7]. To minimize discomfort and instability caused by body movements, stretchable batteries must deviate from existing standardization and accommodate changing forms of flexion and joints in the body. Achieving the necessary softness, elasticity, and stability of stretchable batteries requires substantial modifications to the traditional structures and materials used for essential components, such as the cathode, anode, electrolyte, and separator of conventional batteries [8,9,10,11].

Two approaches have been proposed for realizing stretchable energy storage devices. The first method involves imparting unique structures to hard materials, including wavy types [11,12], island-bridge structures [13], folding structures [14,15], and fiber and spring types [16,17]. However, this type of battery, with a metal-based electrode structure, cannot guarantee stability during repeated stretch/release processes and has limitations in energy density due to its large occupied area within the battery [18]. The second method involves using an elastic polymer as the binder for anode and cathode active materials, polymer electrolytes, and separators [19,20,21]. The binder securely holds the active materials of the cathode and anode on the current collector’s surface and connects them. The use of stretchable binders, such as conductive glue [19] and synthetic polymers based on poly(acrylic acid) or polydopamine [20,21], is gaining attention for preventing delamination and fracturing of electrodes based on ultra-high-capacity silicon active materials. Stretchable gels or polymer electrolytes can serve as both electrolytes and separators, characterized by stable battery operation [9,22]. However, their ionic conductivity is, in general, lower than liquid electrolytes, and the addition of plasticizers for high ion conductivity leads to stability issues that may arise under mechanical deformation due to weak mechanical properties [23]. In addition, poor contacts at the interface between gel electrolytes and electrodes cause weak stability when discharging over many cycles [24,25].

The separator remains essential for providing ion channels between electrodes and preventing shortages of circuits in the battery system. Commercial separators based on polyolefin, such as polypropylene (PP) and polyethylene (PE), exhibit low strain and high tensile strength properties, limiting their application in stretchable batteries [26]. Therefore, developing a stretchable separator is crucial for realizing stretchable energy storage devices.

Polyurethane (PU) consists of isocyanate, polyol, and chain extenders to form a structure with repeating soft segments and hard segments. This structure has excellent elastomer properties. The physical properties of PU depend on the content of the chain extender and the molecular weight of the polyol [27,28]. Various electro-spun fibrous PU separators, such as PU/Al_2_O_3_ [29], PU/graphene oxide (GO) [30], and PU/Poly(vinylidene fluoride) (PVDF) [31], have been reported. In addition, PU has been modified by blending [32], crosslinking [33], and plasticizers [34], showing its potential as a polymer electrolyte. Despite their excellent stretch properties, the electrospinning method has drawbacks, including optimization of separator properties (porosity and pore size), sensitivity to the manufacturing environment (temperature and humidity), and a slow production rate [35]. The solvent-induced phase separation method (SIPS) is another common method to produce porous structures. In this way, the temperature, pressure, and solvent can be controlled to change the morphology. In the case of the non-solvent-induced phase separation (NIPS) method, a non-solvent can provide an additional control parameter to adjust the phase separation in a simpler and faster process, because it may not require the control of pressure and temperature; thus, it allows for control of the pore structure, including the size of the pores [36,37,38]. The non-solvent is typically water or isopropyl alcohol (IPA), which can result in a reduction in the cost. In NIPS, a polymer gel as a film on a substrate is immersed in a non-solvent bath, inducing phase separation of the film into a polymer-rich phase, which becomes the membrane matrix, and a polymer-poor phase, which becomes the membrane pores [37,38].

In this study, we propose the fabrication of a stretchable thermoplastic polyurethane (TPU) separator using the NIPS method with commercially available TPU (see Section 2). Tetrahydrofuran (THF) and IPA were used as a solvent to dissolve TPU and a non-solvent to determine phase inversion, respectively. Both solvents quickly demixed within the cast TPU film, forming a sponge-like pore structure. An excellent mechanical property of fabricated TPU separators can be achieved over repeated cycles of strain/release tests. We also proved the separator’s viability for Li-ion batteries (LiBs) through electrolyte wettability, thermal resistance, air permeability tests, and electrochemical characterizations, which we believe can be nominated as a great candidate for next-generation stretchable separators.

## 2. Materials and Methods

### 2.1. Materials

For this experiment, we fabricated a separator using TPU in granular form (Estane^®^ X595A-11, Mw = 110,000, Lubrizol Corp., London, UK). THF (anhydrous, ≥99.9%, Sigma Aldrich, St. Louis, MO, USA) served as the soluble solvent for TPU, and IPA (99.9%, Samchun Pure Chemical Co., Ltd., Pyeongtaek, Korea) served as a non-solvent. We used the electrolyte as purchased (1.0 M LiPF6 in ethylene carbonate/dimethyl carbonate, Sigma Aldrich, St. Louis, MO, USA). PVDF (MTI Korea, Seoul, Korea), Super P (MTI Korea), and LiCoO_2_, (MTI Korea) were utilized for the fabrication of the cathode, and lithium metal (MTI Korea) was used as the anode.

### 2.2. Fabrication of Stretchable TPU Separator

**Figure 1:**
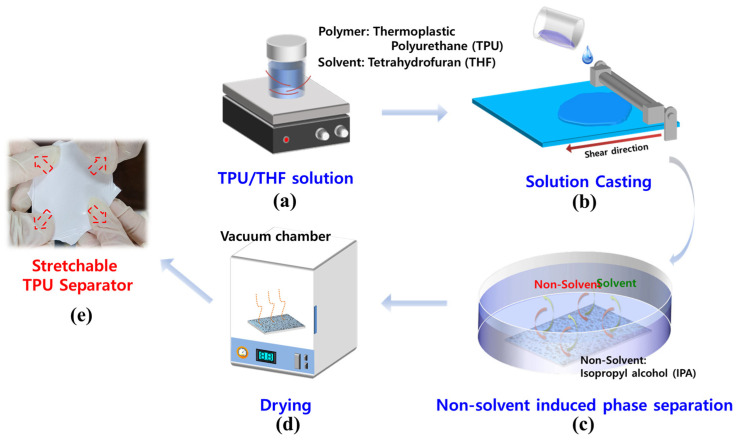
Schematic illustration of fabrication process based on non-solvent-induced phase separation method (NIPS). (**a**) Thermoplastic polyurethane (TPU) was dissolved in tetrahydrofuran (THF) and mechanically stirred at 57 °C for 12 h. (**b**) The TPU/THF solution was casted at a speed of 3 mm s^−1^ with casting thickness of 250 µm. (**c**) The thin layer of TPU/THF solution was submerged in isopropyl alcohol (IPA) as a non-solvent for 24 h. (**d**) The separator was dried in a vacuum oven. (**e**) The TPU separator was fully stretchable.

The fabrication process of stretchable TPU separators, depicted in Figure 1, was accomplished using the NIPS methodology. We dissolved the TPU, in a granular form, into THF through mechanical stirring at 57 °C for 12 h to make TPU solutions of different concentrations (7, 8, and 9 wt%). We then performed bar coating at a speed of 3 mm s^−1^ with a casting thickness of 250 µm, and the final separator thickness was formed with 25 ± 3 µm. To achieve the optimum porous structure in our TPU separators, we carried out the NIPS process at room temperature for 24 h, followed by drying in a vacuum oven at 45 °C for 20 min.

### 2.3. Characterization of TPU Separators

The morphologies of the surfaces and cross-sections of the TPU separators were observed using field-emission scanning electron microscopy (FE-SEM, SUPRA 40VP, Zeiss, Cologne, Germany). A universal mechanical testing machine (AGS-X, Shimadzu, Seoul, Korea) was employed to measure the tensile strength and strain, assessing the mechanical properties of the stretchable TPU separators.

The air permeability of the separators was gauged using a Gurley densitometer (4110N, Thwing-Albert, West Berlin, Germany) and a programmable digital timer (4320DN, Thwing-Albert). We measured porosity using a mercury porosimeter (AutoPore IV 9500, Micromeritics, Haan, Germany) and analyzed the pore size distribution of the separators from SEM images using dedicated software (Image J 1.53k) [39,40].

The thermal shrinkage of the separators was verified in an oven at intervals of 10 °C (from 90 °C to 160 °C) for 30 min. To characterize the thermal stability of the TPU separator, we conducted differential scanning calorimetry (DSC, Discovery DSC 2500, New Castle, DE, USA) under nitrogen flow at a rate of 10 °C min^−1^. The electrolyte’s wettability was evaluated through a contact angle test (L2004A1, Ossila, Sheffield, UK) and an electrolyte uptake test.

The electrolyte uptake property of membranes was calculated by the following formula [39]:

*t* = (*w*_1_ − *w*_0_)/*w*_0_ × 100 (1)

where *w*_0_ is the weight of the dried separator before soaking in the liquid electrolyte and *w*_1_ is the weight of the wet separator after soaking for 1 h and removing the liquid electrolyte on the separator’s surface.

### 2.4. Electrochemical Measurements

The ionic conductivity of the fabricated TPU separator was measured using a potentioStat (ZIVE MP1, WonA Tech, Seoul, Korea). The TPU separator was sandwiched between two stainless steel plates in a coin cell, and the impedance was measured in a frequency sweep from 10^−1^ to 10^5^ Hz, with an amplitude of 1 mV, at room temperature. The ionic conductivity was then calculated using the following formula [39]:

*σ* = *L*/*R*∙*S* (2)

where *L*, *R*, and *S* denote the thickness of the separator, the bulk resistance, and the contact area between the separator and stainless steel plates, respectively. The oxidative stability was evaluated through linear sweep voltammetry (LSV) tests at a scan rate of 1 mV s^−1^ from 3 V to 5 V, performed using a sandwich structure in which the TPU separator was inserted between a lithium metal plate and a stainless steel plate. The coulometric capacity was measured over a voltage range from 2.5 V to 4.2 V, at a certain discharge current density (specified as a C-rate) from 0.2 C to 2.0 C.

## 3. Results

**Figure 2:**
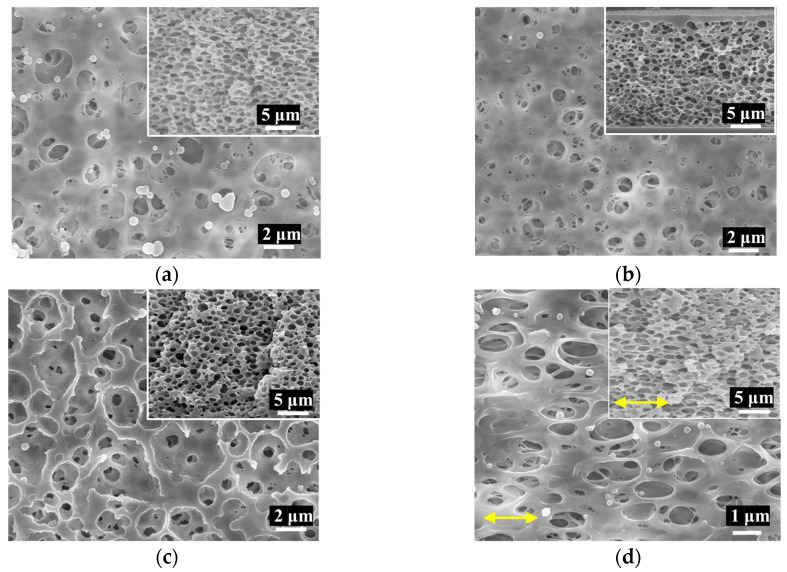
FE-SEM images (at 5000× magnification) of the surface and cross-section (inset) of TPU separators at (**a**) 7 wt% (TPU7), (**b**) 8 wt% (TPU8), (**c**) 9 wt% (TPU9), and (**d**) TPU9 after repeating stretch/release tests with 100% strain over 100 times. Yellow arrows indicate the stretch/release axis.

The FE-SEM analysis of the TPU separator well proves the porosity of the separator, as shown in Figure 2a–c. It revealed variations in the TPU concentrations, which were 7 wt% (TPU7), 8 wt% (TPU8), and 9 wt% (TPU9). Regardless of the concentration variation, we observed great porosity and a sponge-like structure, as observed on the surface and cross-section (insets in Figure 2a–d), but it is important that higher polymer concentrations resulted in a denser, uniformly sized pore structure with thicker polymer walls between the pores. We noted that, even after stretching with 100% strain and 100 cycles, the stretched/released sample TPU9 showed slightly elongated pores, but no structural damage caused by tensile strength, as evidenced in the FE-SEM image shown in Figure 2d.

**Figure 3:**
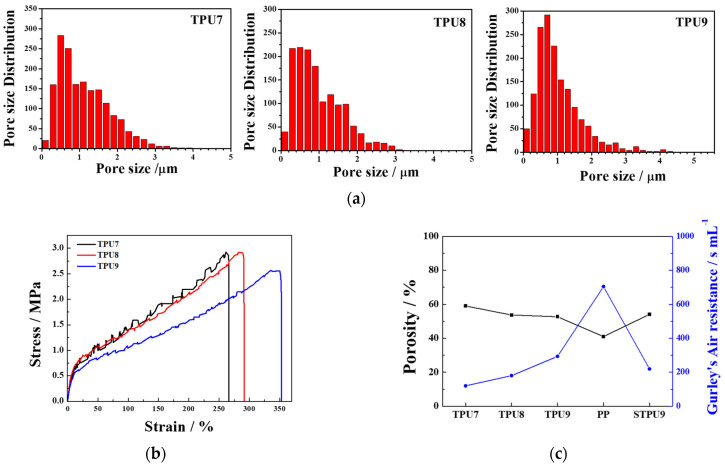
(**a**) Pore size distribution, (**b**) stress–strain curves, and (**c**) porosity measurement and Gurley value of samples TPU7, TPU8, and TPU9. In (**c**), we additionally measured the porosity and Gurley value of PP and TPU9 after repeating stretch/release tests, called STPU9.

Figure 3a shows the measured pore size distribution of the samples TPU7, TPU8, and TPU9. Higher concentrations resulted in predominant pore sizes in a range from about 0.4 μm to 1 μm due to the polymer-rich condition, as the FE-SEM images show in Figure 2a–c. The stress–strain curves of the PP and TPU separators at various polymer contents are depicted in Figure 3b. Notably, the sample TPU9, with a high polymer content, demonstrated a significant elongation at break (352%), likely due to the dense TPU network. The porosity and Gurley’s air resistance in the TPU samples were higher than those in the PP separator. The results, along with the high elongation rate and stability under repeating stretch/release tests and the high porosity found with Gurley’s air resistance test, suggest that NIPS-fabricated TPU separators hold high potential for use in stretchable energy storage devices.

**Figure 4:**
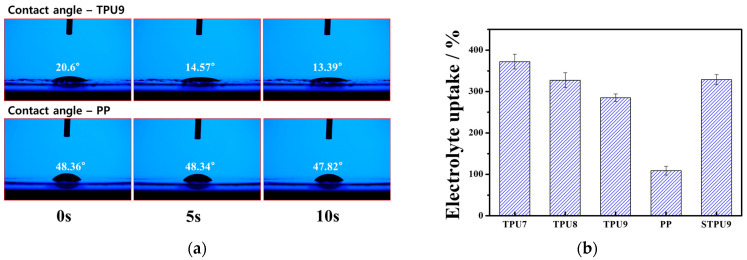
(**a**) Contact angle changes in TPU9 and PP separator, (**b**) electrolyte uptake obtained from TPU separators by TPU content and PP separator.

The wettability of the separators was assessed using contact angle measurement (Figure 4a). Remarkably, the TPU9 sample exhibited a significant decrease in the contact angle from 20.6° to 13.39° within 10 s. In the case of a commercialized PP, minor changes in the contact angle from 48.36° to 47.82° were observed after 10 s. This result shows the superior electrolyte wetting property of TPU9 due to polar groups in polyurethane, as well as the high porosity of TPU9. Figure 4b shows the percentage of electrolyte uptake, which was determined as the remaining amount of electrolyte in each membrane. Markedly higher values were found for TPUs, TPU7 (~372%), TPU8 (~328%), TPU9 (~286%), STPU (~329%), compared to ~109% in the PP separator. As the TPU concentration increased, the mechanical stretchability was enhanced, but the porosity decreased. There was a slight trade-off relation between the strain property and porosity of TPU membranes.

**Figure 5:**
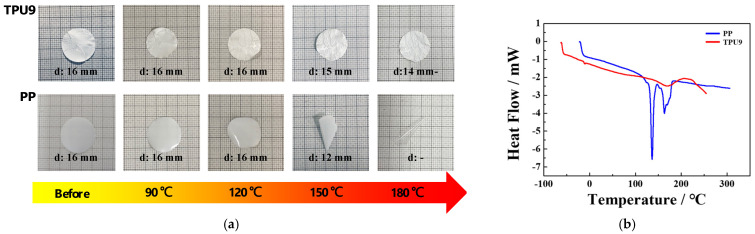
(**a**) Thermal shrinkage and (**b**) temperature-dependent heat flow of TPU9 and PP separators.

The thermal stability of the TPU separator was evaluated through a shrinkage test, gradually increasing the temperature from 90 °C to 180 °C by 30 °C and maintaining each temperature for 30 min (Figure 5a). The membrane structure of the TPU9 sample was well maintained, with its shape only slightly shrinking above 120 °C. In contrast, the PP separator shrank beginning at 120 °C, and substantial shrinkage occurred when the temperature reached 180 °C. As a result of DSC measurement, as shown in Figure 5b, melting of the PP separator occurred near 133–159 °C. In the case of TPU9, the melting occurred in a range of 185–200 °C. The hard segments in the TPU9 contributed to melting at higher temperatures to give better thermal stability.

**Figure 6:**
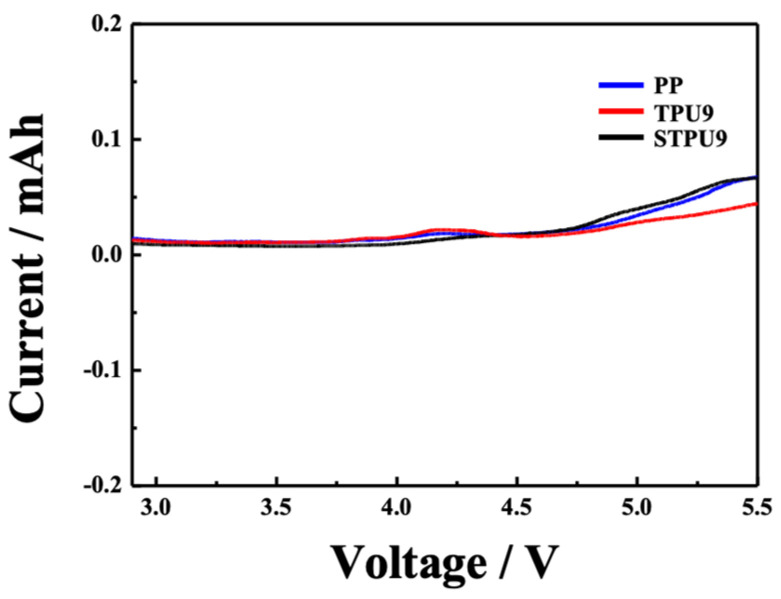
Linear sweep voltammetry (LSV) curves of the cells (stainless steel | separator | Li metal) with the PP, TPU9, and STPU9 separators.

To determine the electrochemical stability of the separators for practical application to LiBs, we conducted LSV measurement of the samples PP, TPU9, and STPU9, as shown in Figure 6. All separators showed about 0.02 mAh increases in current to near 4.3 V, and above 0.05 mAh, the currents rose to near 4.7 V. Notably, STPU9, subject to repeating stretch/release tests, exhibited high electrochemical stability compared to the commercial PP separators.

**Figure 7:**
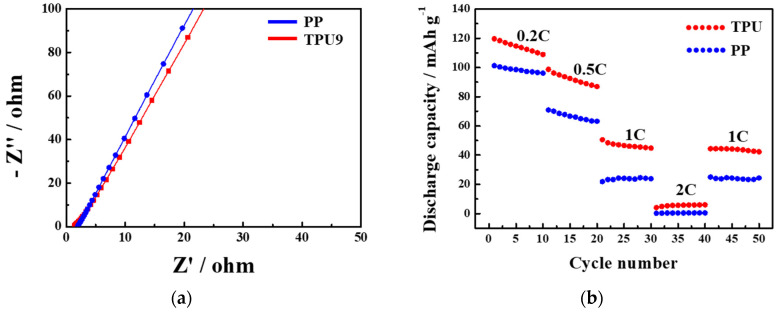
(**a**) Impedance measurement of the cells (stainless steel|separator|stainless steel) with as-prepared separators at room temperature. (**b**) Comparison between capacities of the Li/LiCoO_2_ cells with as-prepared PP and TPU9 separators at different C-rates.

The ion conductivity of the PP and TPU9 separators was verified by measuring impedance, as shown in Figure 7a. The ion conductivity levels of PP and TPU9 were calculated as 4.52 × 10^−4^ S cm^−1^ and 5.58 × 10^−4^ S cm^−1^, respectively, indicating successful ion migration pathways for high ionic conductivity. Lastly, Figure 7b presents the discharge capacity of the PP and TPU9 separators over cycles at 0.2 C–2 C. The TPU9 separator demonstrated good rate-capability, attributed to its excellent porosity and electrolyte wettability. Moreover, the 1 C rate of the reverse cycle closely matched the initial 1 C rate discharge capacity, suggesting its potential applicability in LiBs.

## 4. Conclusions

Stretchable TPU separators, exhibiting an elongation of 352%, were successfully fabricated via the NIPS method. These separators revealed controlled porosity, determined by the TPU concentration and the appropriate use of solvent and non-solvent. The TPU separator, upon undergoing repeated stretch/release tests, demonstrated admirable mechanical and electrochemical stability up to 4.5 V, as evidenced by measurement of the stress–strain curves, FE-SEM analysis, LSV, and coulombic capacity tests in the C-rate from 0.2 C to 2 C. This suggests that separators within LiBs can maintain stability even after extreme motion and deformation. Furthermore, the TPU separator boasts an excellent Gulrey value, exceptional electrolyte wettability, and remarkable thermal stability, enduring temperatures up to approximately 180 °C. In addition, the LiB assembled utilizing the TPU separator delivered a commendable C-rate capability performance, even when compared with PP separators. In summary, the proposed TPU separators possess competitive characteristics and exhibit good performances, positioning them as viable candidates not only for stretchable LiBs, but also for other energy storage devices.

## Data Availability

The data presented in this study are available upon request from the corresponding author.
